# Fusion of UAV-based infrared and visible images for thermal leakage map generation of building facades

**DOI:** 10.1016/j.heliyon.2023.e14551

**Published:** 2023-03-15

**Authors:** Soroush Motayyeb, Farhad Samadzedegan, Farzaneh Dadrass Javan, Hamidreza Hosseinpour

**Affiliations:** a School of Surveying and Geospatial Engineering, College of Engineering, University of Tehran, Tehran, Iran; b Faculty of Geo-Information Science and Earth Observation (ITC), University of Twente, 7522 NB, Enschede, the Netherlands

**Keywords:** Improving energy efficiency, UAV, Point cloud, Fusion, Thermal leakage, Regioin-based segmentation

## Abstract

To make the best use of available energy resources and reduce costs, improving the energy efficiency of buildings has become a critical issue for the construction industry. Today, developing a three-dimensional model of the energy consumption rates in buildings based on thermal infrared images is essential to visualize, identify and increase energy efficiency. The purpose of this study is to suggest a methodology for generating a thermal leakage map of building facades utilizing the fusion of thermal infrared and visible images captured by Unmanned Aerial Vehicles (UAVs). In general, the proposed method involves three basic steps: the generation of thermal infrared and visible dense point clouds from the building’s facade using Structure from Motion (SfM) and Multi-View Stereo (MVS) algorithms; the fusion of visible and thermal infrared dense point clouds using the Iterative Closest Point (ICP) algorithm to overcome thermal infrared point cloud constraints; the use of edge extraction and region-based segmentation methods to determine the location of the thermal leakage of building facade’s. To that end, two datasets obtained for separate building facades are used to assess the proposed strategy. The results of the data analyses for the extraction of the desired components and determination of thermal leakage locations on the building facets provided a Precision and Recall score of 87 and 90% for the first dataset and 87 and 88 for the second dataset. Examining the outcomes of calculating thermal leakage zones indicates improving Precision and Recall.

## 1 Introduction

Over the years, improving the energy efficiency of buildings has become a global priority [1]. With buildings accounting for one-third of overall energy consumption, improving their energy efficiency can reduce greenhouse gas emissions by one-third from an environmental standpoint and can also be economically beneficial to the building occupants [2]. As a result, due to such concerns as climate change, rising energy costs, and energy performance standards, justifying and rationalizing energy consumption in buildings have become a priority for most developed and developing countries [3,4].

**Fig. 1:**
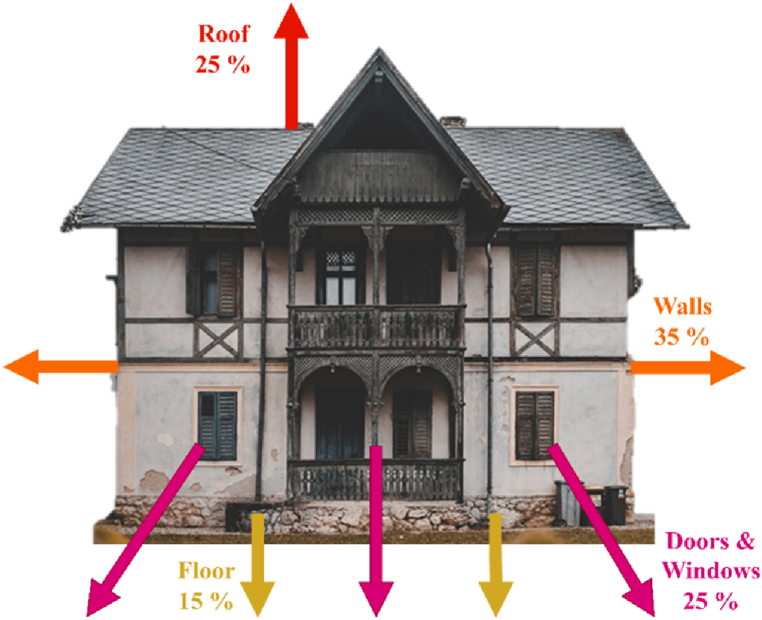
Illustrates the extent of thermal leakage in various areas of the building. (Colored).

In the building sector, energy inspections are conducted to discover faults or cracks, air leakage sources, moisture, cracks, and the suitability of windows to insulate and to evaluate the air conditioning system performance [[5], [6], [7]]. Thermal leakage areas are considered significant deficiencies in the building’s isolation and they mainly occur through thermal bridges or excessive heat loss, air leakage, and the lack or improper use of thermal insulation in building components [8,9]. The percentages of thermal leakage in various parts of a building are depicted in Fig. 1.

Based on the results shown in Fig. 1, the building facades account for most thermal leakage.^1^ Therefore, producing a thermal leakage map of the building facades can be a necessary step in achieving appropriate information on the building’s energy performance.

Thermography is a cost-effective, non-destructive convenient method widely used not only for interpretive and numerical measurements of building thermal leakage [10,11] but also for detecting abnormalities [12] and reporting on the existing energy efficiency of buildings [13,14].

As two-dimensional thermographic images are insufficient for analyzing the exact location of thermal problems, it seems necessary to use a three-dimensional thermal model of a building’s energy status [15] in order to visualize energy performance and to get the possibility of simultaneous estimation and analysis. Thus, demonstrating a building’s present energy status entails two general steps: a) geometric 3D modeling and b) localizing, measuring, and evaluating the thermal leakage [16].

In recent years, computer-vision and photogrammetry activists have taken a keen interest in the usage of UAVs as a result of advancements in the technology of sensors used in UAVs and their ability to provide information with high resolution and accuracy. Moreover, using UAVs increases the flexibility of data collecting and enables the combination of several sensors for data acquisition [17]. In this regard, the advantages of utilizing UAVs include their high maneuverability in urban environments, their ability to collect overlapping images from various viewing angles, and their ability to get to the proximity of an object. Therefore, the aforementioned benefits have all made UAV systems a standard and dependable platform for collecting data to produce three-dimensional models based on image processing algorithms such as Structure from Motion (SfM) [18,19]. Additionally, by utilizing orthophoto mosaics obtained via UAV-photogrammetry, it is possible to directly and accurately measure distances, angles, and planimetric coordinates [20].

Moreover, due to UAVs capability to be equipped with various sensors, including inexpensive and lightweight thermal cameras, many activists in this field have considered UAVs as cutting-edge technology for quantitative and qualitative analysis of thermal properties and air inspection of buildings. Additionally, it is also possible to get high-quality thermal infrared images of a structure from various angles utilizing UAVs. This not only generates a thermal 3D infrared visualization and identifies thermal bridges but also assists in quantifying the thermal transmittance (U-value) of wall and ceiling surfaces through the use of thermal infrared orthophoto mosaic.

In this paper, by employing the RGB point cloud as the three-dimensional geometric reference, the thermal point cloud drawbacks, such as low density and poor texture, can be overcome by fusing visible and thermal images at the point cloud level [21,22]. The goal of this study is thermal leakage map generation of building facades by using visible and thermal infrared images obtained by the UAV.

### 1.1 Related works

Considering the low spatial resolution and contrast of thermal infrared images and the use of a narrow field of view (FOV) in infrared cameras [21], obtaining a relatively noisy and low-density point cloud is one of the challenges of this research. One possible option is to map thermal images onto the visible point cloud. The point cloud derived from visible images with high spatial resolution contains detailed geometric information for the quantitative and qualitative study of various building components. Thus, quantitative and qualitative thermal evaluations can be performed by mapping the texture of thermal images onto a visible point cloud as a geometric reference. In the following, studies on thermal texture mapping to visible point cloud are reviewed.

Lagüela et al. [23] mapped the thermal texture onto the 3D scanner laser point cloud using epipolar geometry by calculating the homography transformation between each thermal image and the laser scanner point cloud. Son et al. [24] thermal images have been mapped onto a three-dimensional point cloud generated from visible images by calculating the best transformation matrix through the Random Sample Consensus (RANSAC) algorithm. López-Fernández et al. [25] to determine the amount of thermal leakage inside the building, a mobile mapping system equipped with a thermal sensor was used to generate a thermal orthophoto mosaic. By space resection between the thermal images and the point cloud, the thermal texture was mapped onto the point cloud. Han et al. [26] used space resection to map the thermal texture to a visible three-dimensional model utilizing a UAV equipped with thermal and visible cameras and simultaneous imaging, which could generate the same exterior orientation parameters for images.

The usual methods of homography transformation and space resection are used to map a thermal texture onto three-dimensional data. Nevertheless, the approach to using space resection has at least three drawbacks: The first is the time-consuming processing because when thermal video data is used, each image’s space resection must be performed independently. The second issue is the difficulty to determine the minimum control points in weakly textured thermal images, which can impair the final orientation’s accuracy. Thirdly, the 3D model’s mosaic of thermal images is prone to exhibit discontinuities in overlapping areas. The proposed solution is the fusion of thermal and visible images.

Dadras Javan et al. [21] generated a three-dimensional high spatial resolution thermal textured model using the Iterative Closest Point (ICP) algorithm to fuse visible and thermal point clouds derived from UAV images. Lin et al. [27] produced visible and thermal point clouds independently using the SfM algorithm for both thermal and visible data for large-scale thermal mapping. Finally, they fused the two datasets using the ICP technique to support the geo-referenced data. Dahaghin et al. [22] used the ICP algorithm to produce a spatial high-resolution point cloud from the roof of the building by fusing the visible and thermal point clouds. Grechi et al. [28] applied the fusion of visible and thermal infrared images at the level of point cloud for producing the point cloud of rock masses prone to failure of the ICP algorithm was applied. The following discusses methods of determining the locations of thermal leakage.

Hoegner et al. [29] identified the thermal leakage areas on the three-dimensional textured model of the building by utilizing segmentation algorithms and comparing the intensity of the grayscale values in the desired segments with the average value of the grayscale values. Gorzalka et al. [30] determined the quantity of thermal leakage using the object-oriented building model and U-value concept following a three-dimensional reconstruction based on thermal and visible images of the building.

Rakha et al. [31] used UAV images to swiftly assess the building’s energy performance and generate an aerial map of thermal leakage. The areas of the building affected by thermal leakage were inspected using computer vision approaches such as edge detection and the Region-Growing segmentation algorithm. In evaluating the thermal leakage zones using two machine learning criteria, that is, Precision and Recall, they got 76% and 74%, respectively. In Kakillioglu et al. [32] thermal leakage was detected using a Region-Growing segmentation method and morphological operators. In assessing the detection of thermal leakage zones using Precision and Recall criteria, 91%, and 89% values were obtained for the handheld camera dataset, and 34% and 71% for the UAV dataset respectively.

Zhong et al. [33] developed a heat-infrared thermal saliency map for detecting thermal leakage in district heating systems based on the degree of Saliency to improve thermal target detection. The resulting saliency map was then merged with adaptive target segmentation with maximizing entropy to enable automatic detection of possible thermal leakage targets. Sledz et al. [34] used a circular Laplacian of Gaussian (LoG) detector to identify high-temperature locations in thermal orthophoto mosaics. Then using segmentation and classification algorithms, thermal abnormalities were localized. In Baktykerey et al. [35], for detecting thermal leakage and producing a mask for filtering out areas of the highest temperature, the images were converted from infrared to HSV color space. Vorajee et al. [36] showed that when the results of several image segmentation algorithms for detecting the location and amount of thermal leakage were compared, the region-based segmentation algorithm would produce the best results for determining the areas of thermal leakage.

The method proposed in the present study generates an actual three-dimensional thermal model of the building. Additionally, by utilizing UAVs, the challenges and drawbacks associated with traditional thermographic methods, such as a lack of access to inaccessible and out-of-sight regions for evaluation and data gathering, are resolved. Moreover, by utilizing data from UAVs and aerial imagery, a broader area of buildings may be evaluated in less time. All this provides the possibility to use the ICP method along with a region-based segmentation algorithm to fusion thermal infrared and visible images and to detect and determine thermal leakage locations on the building facade.

The possible outcomes of the study can be summarized as follows: comprehensive visualization of the thermal condition of building facades, improvement and removal of limitations associated with thermal infrared point cloud (low density and noise), orthophoto mosaic produced by fusing thermal infrared and visible point clouds, automatic extraction of building facade components, and finally identification and determination of areas of thermal leakage in the building facade.

The paper is organized into five sections. Section 2 describes and discusses the methodology. The results of the proposed approach are presented in Section 3, the discussion in section 4, and the conclusions and recommendations for future studies in section 5.

## 2 Methodology and materials

**Fig. 2:**
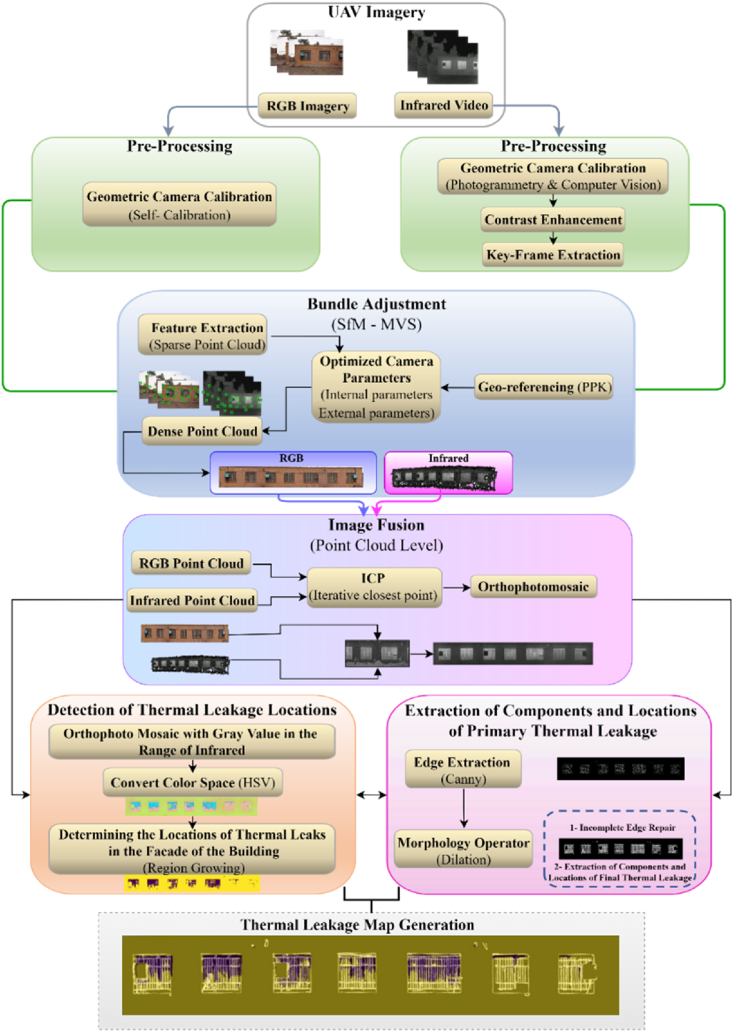
Flowchart of the proposed method. (Colored).

The proposed method for thermal leakage determination is composed of three main steps (Fig. 2). The first is pre-processing and generating a precise and dense three-dimensional model for the existing structure’s facade. The second step involves fusing visible and thermal infrared point clouds, and the third consists of extracting building facade components for recognizing, detecting, and determining areas of thermal leakage in the building’s various facades.

Based on the flowchart in Fig. 2, after completing the necessary pre-processing on thermal and visible images, such as keyframes extraction and geometric camera calibration, the thermal and visible point cloud were generated using bundle adjustment (SfM and Multi View Stereo (MVS)) algorithm [37]. Then, utilizing the Post-Processing Kinematic (PPK) module and accurate Global Positioning System (GPS) navigation information, thermal infrared and visible point clouds were geo-referenced.

To overcome the constraints of thermal infrared point cloud, such as low density and relative noise, visible and thermal point clouds were fused using the ICP algorithm. As a result, the fused point cloud contained both visible and thermal information with high spatial resolution and density. Additionally, the fused point cloud was employed to generate a three-dimensional model and an orthophoto mosaic of the building facade.

Finally, a thermal leakage map was generated by extracting the building facade components using the canny edge extraction operator and segmenting the thermal leakage area through the use of the region-growing segmentation technique.

### 2.1 Study area

**Fig. 3:**
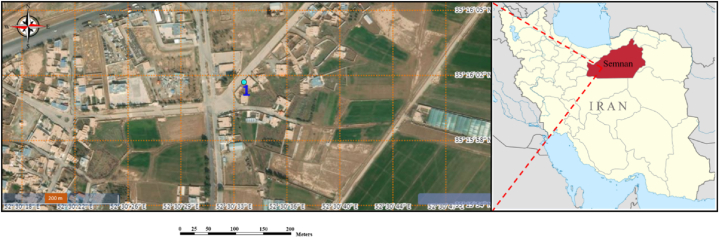
Study area. (Colored).

Thermal infrared and visible data on the building facades were collected at the Pedar Salar mansion ecotourism residence in Aliabad village, Aradan-Garmsar city, Semnan province, Iran, with longitude coordinates of 52.509185 and latitude coordinates of 35.266896. In this paper, two datasets of visible images (first and second datasets, respectively: 46 and 24 images) and thermal infrared video (first and second datasets with a rate of 30 frames per second, respectively: 70 s and 35 s) were collected from the two facades of the studied building. Fig. 3 depicts the research region.

### 2.2 UAVs and thermal infrared and visible cameras

In this study, to collect the required data, a vertical flight UAV equipped with thermal infrared and visible cameras was utilized. Additionally, in order to control and guide the UAV using telecommunications equipment, a computer, with Mission Planner software was utilized. The flight path was designed to collect data perpendicular to the building facades at a distance of 11 m and a flight height of 1.70 m from the building facades. In this paper, the imaging scale for visible data is 1:314 and for thermal infrared data, it is 1:440, based on the focal length and distance from the building facade. In addition, the UAV flew at a slow speed, and images of the building facades were collected with an 80% overlap. Table 1 contains more detailed information about the UAV that was employed.

**Table 1:** Technical characteristics of UAV. (Colored).

Parameters	Values	UAV
Flight duration	30–40 min	
Flight altitude	300 m	
Maximum weight	6.5 kg	
Dimensions	0.9 m	
Cruise speed	50 km/h	
Wind resistance	35 km/h	

The MC1-640s thermal infrared camera of KeiiElectro Optics Technology at 30 frames per second and the Sony ILCE-7RM4 visible camera was selected for data collection. Table 2 represents the technical specifications of thermal infrared and visible cameras.

**Table 2:** Thermal infrared and visible cameras technical specifications. (Colored).

Camera	Parameters	Values	
Visible	Resolution	9504 × 6336	
	Focal length	35 mm	
	Sensor dimensions	35.7 × 23.8 mm	
	Detector type	CMOS	
Thermal Infrared	Resolution	480 × 640	
	Focal length	25 mm	
	Sensor dimensions	0.017 mm	
	Thermal sensitivity	0.03–30C°	

### 2.3 Pre-processing

#### 2.3.1 Visible and thermal infrared camera geometric calibration

**Fig. 4:**
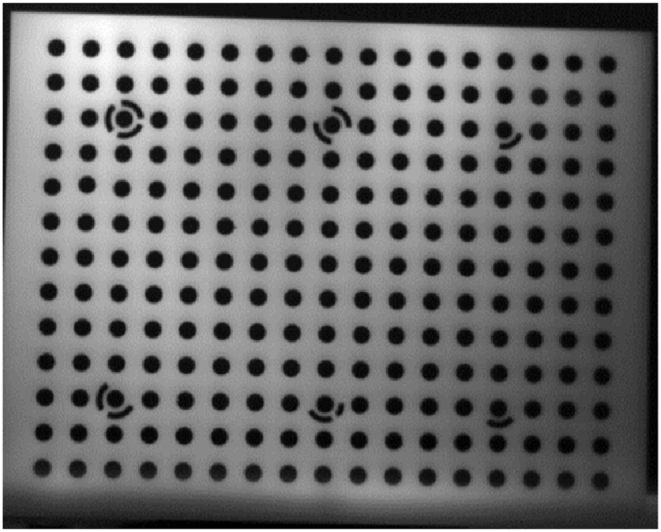
The pattern of calibration. (Colored).

In this study, the self-calibration [38] approach was applied to perform geometric calibration on the visible camera. Additionally, this study required the geometric calibration of the thermal infrared sensor, which was performed using a combination of photogrammetry and computer vision techniques. A rectangular plate with hollow circles was used as the calibration pattern since due to the circle’s flexible geometry, it is possible to identify the ideal ellipse in images captured from the calibration pattern and get satisfactory results [39,40]. The calibration pattern utilized in this study is represented in Fig. 4.

As thermal infrared cameras have low spatial resolution and low contrast, circular targets appear as ellipsoids in the image. Therefore, to solve the issue, the Hough Transform algorithm (HT) [41] was utilized to accurately fit and extract the two-dimensional coordinates of the focal center of ellipsoid targets in the image space. The HT provides the ellipse using the ellipse’s central parameters and does not require the complete extraction of the ellipse’s circumferential lines. The geometric calibration parameters of the thermal infrared camera are then computed using the collinearity equation provided by Eqs. (1), (2), and the relationship between 2D and 3D space is established.

(1) $$x_{a}=x_{p}-c\frac{r_{11}\left(X_{A}-X_{O}\right)+r_{21}\left(Y_{A}-Y_{O}\right)+r_{31}\left(Z_{A}-Z_{O}\right)}{r_{13}\left(X_{A}-X_{O}\right)+r_{23}\left(Y_{A}-Y_{O}\right)+r_{33}\left(Z_{A}-Z_{O}\right)}$$

(2) $$y_{a}=y_{p}-c\frac{r_{12}\left(X_{A}-X_{O}\right)+r_{22}\left(Y_{A}-Y_{O}\right)+r_{32}\left(Z_{A}-Z_{O}\right)}{r_{13}\left(X_{A}-X_{O}\right)+r_{23}\left(Y_{A}-Y_{O}\right)+r_{33}\left(Z_{A}-Z_{O}\right)}$$

where *c* stands for the focal length, *x*_*a*_, *y*_*a*_ are the image coordinates, *x*_*p*_, *y*_*p*_ represent the principal point coordinates, *X*_*0*_, *Y*_*0*_, *Z*_*0*_ are the projection center coordinates, *X*_*A*_, *Y*_*A*_, *Z*_*A*_ stands for the object space coordinates, and *r* designates the rotation matrix element. Additionally, the lens distortion parameters are iteratively estimated using Brown equations [42] (Eqs. (3), (4)).

(3) $$x^{\prime }=x\left(1+k_{1}r^{2}+k_{2}r^{4}+k_{3}r^{6}+p_{2}\left(r^{2}+2x^{2}\right)+2p_{1}xy\right)$$

(4) $$y^{\prime }=y\left(1+k_{1}r^{2}+k_{2}r^{4}+k_{3}r^{6}+p_{1}\left(r^{2}+2y^{2}\right)+2p_{2}xy\right)$$

The variables *x'* and *y'* are the rectified image coordinates, *k* stands for radial distortion coefficients of the lens, *p* represents tangential distortion coefficients of the lens, and *r* is the radial distances from the perspective point image on the image plane in Brown equations.

#### 2.3.2 Keyframes extraction

In this paper, due to spatial resolution issues, low contrast as well as the poor texture of thermal images and building facades, a contrast enhancement step was performed on raw thermal video frames. Contrast enhancement is used to improve the extraction of corresponding features from images and to ensure that the standard baseline check stage in keyframe extraction is correctly done. As a result, the contrast enhancement method described in research [43] was adopted to enhance the contrast of thermal infrared images used in the keyframe extraction step. This method which is called the multiscale top-hat transform [44] uses two proportionate and flat geometric structural elements to increase the detail of thermal infrared images by improving global entropy. In thermal infrared images, it also retains normal brightness and enhances the contrast while reducing distortion in the output image.

Keyframes extraction from the video is a necessary pre-processing step for increasing the geometric accuracy and computing speed of the thermal infrared dense point cloud generation. Keyframe extraction in this study entailed determining the amount of blurriness and the baseline of overlap, checking the degeneracy conditions, and extracting the keyframes.

One of the fundamental drawbacks of video frame processing is the presence of low-quality radiometric frames, which appear in the frames as Motion Blur [45]. This effect impairs photogrammetry and video-grammetry algorithm processing [45]. Given that keyframes extraction is a vital step in the process of 3D reconstruction of the video, the idea of recognizing and eliminating frames with blurriness had been suggested [[46], [47], [48]]. In this study, to identify and remove blurred frames, the Fast Fourier Transform metric (FFT) [49,50] was used.

After analyzing the radiometric quality and removing the blurred frames, appropriate geometric information was extracted from high-radiometric (non-blur) video frames to improve geometric accuracy and reduce the number of computations required for the 3D reconstruction process. This step involved determining the standard baseline between successive frames by evaluating the overlap between the features extracted from the sequential frames and the Geometric Robust Information Criteria (GRIC) [51] to achieve the best geometry for the frames during the 3D reconstruction process.

In this stage, to determine the standard baseline between the frames for keyframes extraction, the first frame from the set of frames was chosen as the keyframe. Additionally, features were extracted using the SIFT algorithm [52]. The proportionality coefficients of the corresponding key points between the two frames were then calculated by Eq. (5). This coefficient indicates the ratio of the number of key points that correspond between two frames to the number of key points extracted from the keyframe [53]. Besides, to calculate the search range for frames with standard baselines using the proportionality coefficient of key points, the two minimum and maximum permissible thresholds of 0.6 and 0.8 were chosen.

(5) $$R_{c}=\frac{T_{c}}{T_{f}}$$

In Eq. (5), *R*_*C*_ indicates the proportionality coefficient of the corresponding key points, *T*_*C*_ is the number of corresponding key points between the two frames, and *T*_*f*_ stands for the total number of key points extracted from the first keyframe. Following an examination of the standard baseline, the frames would be studied to analyze degeneracy conditions.

The fundamental matrix is used to investigate the camera’s overall structure in various locations and the relationship between the corresponding features in the two frames. However, it is impossible to determine the camera’s location in degeneracy conditions. In degeneracy conditions, two important factors are motion and structural degeneracy [54].

In degeneracy states, the image pair is matched using a homography matrix. As a result, comparisons between the homography and fundamental matrixes are made to assess degradation conditions using the GRIC criterion as the ideal selection criterion [54]. Eq. (6) defines the GRIC criterion as follows:

(6) $$GRIC=\sum \rho \left(e_{i}^{2}\right)+\left(\lambda_{1}nd+\lambda_{2}k\right)$$

In Eq. (6), parameter *n* denotes the number of corresponding inlier properties extracted in the fundamental matrix and homography solution, *e*_*i*_ indicates the residual vector. The numerical value of *k* equals the motion model parameters for the homography and fundamental matrices, and d stands for the dimensions of the model structure.

Finally, using the criteria stated in Eq. (7), a frame will be chosen as the keyframe if the fundamental matrix model between the two frames has a numerical value less than the homography matrix model in terms of the GRIC criterion.

(7) $$f_{G}\left(i,j\right)=\frac{GRIC_{F}\left(i,j\right)-GRIC_{H}\left(i,j\right)}{GRIC_{H}\left(i,j\right)}$$

### 2.4 Thermal infrared and visible point cloud generation

Various methods in photogrammetry and computer vision, such as object bundle adjustment and SfM, have been presented to generate a point cloud and a three-dimensional model based on a set of input images. Image-based reconstruction methods such as SfM and MVS are used to generate photogrammetry outputs with a high spatial resolution (point cloud, 3D model, and orthophoto mosaic) from inexpensive non-metric camera images mounted on UAVs with overlap forward and sidelap (75–80%).

Processing the obtained images begins with automatic keyframes extraction [37,55]. Then, to describe each point, the features extracted by a multidimensional descriptor such as SIFT with 128 vectors are defined. As the matching of extracted features is based on the descriptor’s maximum multidimensional probability [37] and the Outlier rejection criterion [56], using the bundle adjustment, the camera’s interior and exterior orientation parameters are simultaneously solved to generate a sparse point cloud [57].

The reconstructed model is converted to an object coordinate system (absolute orientation) via control points or by estimating the camera position using the UAV based on GPS (using the PPK approach). This information is then entered into the model, and the second bundle adjustment is carried out [58].

The MVS algorithm can be used to increase the density of the point cloud, which generates a depth map for image pixels based on their image stability in the oriented bundle adjustment output block [55,59,60]. Following this stage, a dense image-based point cloud is obtained, and other photogrammetry products such as orthophoto mosaics can be produced using these point clouds.

### 2.5 Thermal infrared and visible point cloud fusion

Various challenges are associated with producing a three-dimensional point cloud from thermal infrared images acquired by UAVs using the SfM method. For instance, the low spatial resolution and narrow FOV of thermal infrared sensors, along with blurred edges and a lack of texture in many sections of the image, can result in the failure of the SfM algorithm or may produce suboptimal results [61]. In this paper, to address these deficiencies and limitations, the fusing of visible and thermal infrared point clouds based on the image was applied.

To overcome the limitations of the thermal infrared point cloud, including the low-density point cloud, which is resulted from the low spatial resolution of thermal infrared images, it is necessary to fuse the visible and thermal infrared point clouds. In the study, radiometric information was mapped from the nearest three-dimensional point in the thermal infrared point cloud to its equivalent point in the visible point cloud using the ICP algorithm [62].

### 2.6 Extraction of building facade components

Following the fusing of visible and thermal infrared point clouds, an orthophoto mosaic was generated utilizing the fusion point cloud. The orthophoto mosaic described earlier was used to extract the components of the building facade in this paper. The first step was to generate the orthophoto mosaic temperature histogram, which was utilized to establish two appropriate thresholds for extracting components and areas associated with building facade thermal leakage. The histogram depicts the grayscale value distribution of the image pixels. Thus, the orthophoto mosaic histogram presents the distribution of grayscale values in orthophoto mosaic pixels, as well as the numerical value of the temperature assigned to each pixel.

The first stage involved calculating two thresholds based on the temperature distribution to examine locations prone to thermal leakage. Rather than defining a predefined threshold, two specific thresholds for use in the edge extraction step were chosen for the initial extraction of facade components and areas prone to thermal leakage. The extraction of acceptable thresholds was done by calculating the grayscale value histogram associated with the temperature distribution. It was divided into five distinct groups empirically and through trial and error.

**Fig. 5:**
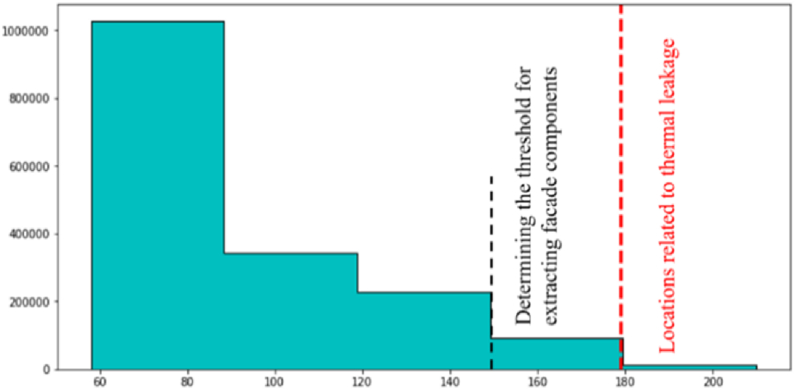
Determining two extraction thresholds. (Colored).

The relevant thresholds for extracting facade components and thermal leakage locations based on histogram classification are shown in Fig. 5. As Fig. 5 shows, while the threshold for finding thermal leakage locations involves investigating candidate areas for thermal leakage, the threshold for extracting facade components includes the study of adequate temperature change in candidate areas.

The horizontal axis in Fig. 5 represents the intensity of the orthophoto mosaic pixel’s grayscale values. The two thresholds designated as the optimal threshold values are input to the step of edge extraction. In this stage, the canny edge extraction operator was used to extract the initial components of the facade and locations of thermal leakage. Following the extraction of the initial edges, the dilation morphology operator was employed to enhance the edges and extract the facade components and locations of thermal leakage. Finally, all facade components and regions susceptible to thermal leakage were completely extracted.

### 2.7 Thermal leakage map generation

According to studies on stochastic segmentation algorithms [36], for determining the areas of thermal leakage, region-based methods outweigh edge-based and threshold-based methods. Thus, in this paper, thermal leakage locations were selected using a region-based segmentation technique.

In computer vision, image segmentation is a technique for dividing a digital image into multiple parcels or regions. The primary goal of image segmentation is to separate the image into several regions with modest grayscale value changes. In other words, there is nothing in common between the regions [63].

The Region-Growing segmentation technique was employed in this paper to determine the locations of thermal leakage. The Region-Growing algorithm is a method for grouping pixels or image portions into more extensive regions depending on predefined growth parameters. This method works by starting with a collection of “seed” points in the image areas and nearby pixels with preset seed-like features (such as a specific range of grayscale values or color intensity). This algorithm’s performance is based on the fact that pixels nearby have similar grayscale value intensities.

Then an initial set of small regions are merged repeatedly based on similarity criteria. When a part stops growing, another seed pixel is selected that does not yet belong to any area, and the algorithm steps start again from the beginning. The algorithm will stop if any pixel no longer meets the inclusion criteria in that part [63].

**Fig. 6:**
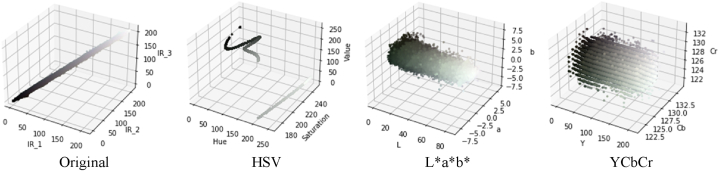
Illustrates the orthophoto mosaic pixel distribution in various color spaces. (Colored).

The purpose of this paper was to segment the locations associated with thermal leakage. In this case, because the distribution of the desired image pixels in the HSV color space would provide a better separability for segmentation, seed pixels were selected from the HSV color space saturation parameter. In Fig. 6, the distribution of orthophoto mosaic pixels in the HSV, L * a * b *, and YCbCr color spaces were investigated to determine the seed pixels for the segmentation algorithm.

The distribution of orthophoto mosaic pixels in various color spaces is depicted in Fig. 6. As can be seen in Fig. 5, for extracting areas with higher temperatures, thermal infrared orthophoto mosaic pixels in HSV color space provide more separability for image segmentation and assessment of thermal leakage locations. As a result, seed pixels were chosen in this study from the HSV color space saturation parameter.

### 2.8 Criteria for evaluating the proposed approach for thermal leakage map generation

To assess the proposed method for determining thermal leakage areas in this paper, two well-known machine learning evaluation criteria, precision, and recall, were applied. The precision criterion is mainly concerned with the proposed algorithm’s proper detection of thermal leakage locations. In contrast, the recall criterion is primarily concerned with data that have been correctly identified as thermal leakage locations by the suggested approach.

The precision criteria are the ratio of true positives (*T*_*P*_) to all positives plus false positives (*F*_*P*_). Additionally, the recall criterion can be represented by dividing true positive samples by positive samples plus false negative (*F*_*n*_) pieces. Also, both assessment criteria range from zero to one, and the closer the output value is to one or one hundred percent, the more acceptable the proposed method’s performance for both precision and recall criteria will be. Precision and recall criteria are defined as follows using Eqs. (8), (9):

(8) $$\text{Precision}=\frac{Tp}{Tp+Fp}$$

(9) $$\text{Recall}=\frac{Tp}{Tp+Fn}$$

The variables used to define the precision and recall criterion in the above equations are as follows:

• True Positive (*T*_*P*_): The number of pixels predicted correctly by the proposed algorithm.
• False Positive (*F*_*P*_): The number of pixels mispredicted by the proposed algorithm.
• False Negative (*F*_*n*_): The number of pixels we expected the algorithm to predict but it did not.

## 3 Results

In this paper, to meet the objectives, an approach was introduced and evaluated in the Python programming language to determine the thermal leakage locations on the building facades. This section describes the equipment and data that were used to carry out the research. The following sections describe and discuss the results of the study. They include pre-processing sections on the geometric calibration of thermal infrared and visible cameras, contrast enhancement of thermal infrared images, and keyframes extraction from thermal infrared video. Next, the production of visible and thermal infrared point cloud, the fusion of visible and thermal infrared point cloud, the production of photogrammetry products such as orthophoto mosaic, the extraction of building facade components, and eventually determination of sites linked to building facade thermal leakage carried out.

### 3.1 Thermal infrared and visible camera geometric calibration

In this paper, to calibrate the visible camera geometrically using the self-calibration method, 46 overlap images (the first visible dataset) of the building facade were employed. Through self-calibration, parameters of interior orientations and lens distortions were computed. The correlation matrix and calibration parameters of the visible camera (first visible dataset) in terms of pixel measurement unit are reported in Table 3.

**Table 3:** Visible camera calibration parameters for first visible dataset (pixel).

Parameters	Value	Error	*K*_*1*_	*K*_*2*_	*P*_*1*_	*P*_*2*_
*F*	9240					
*K*_*1*_	0.0357	8.9 e−05	1.00	−0.72	−0.14	−0.14
*K*_*2*_	−0.1800	0.00013		1.00	0.03	−0.06
*P*_*1*_	0.0009	1.5 e−05			1.00	0.04
*P*_*2*_	−0.0006	1.5 e−05				1.00

In Table 3, *F* indicates the focal length, *K*_*1*_ and *K*_*2*_ stand for the radial distortion, and *P*_*1*_ and *P*_*2*_ represent the tangential distortion of the visible camera lens.

**Fig. 7:**
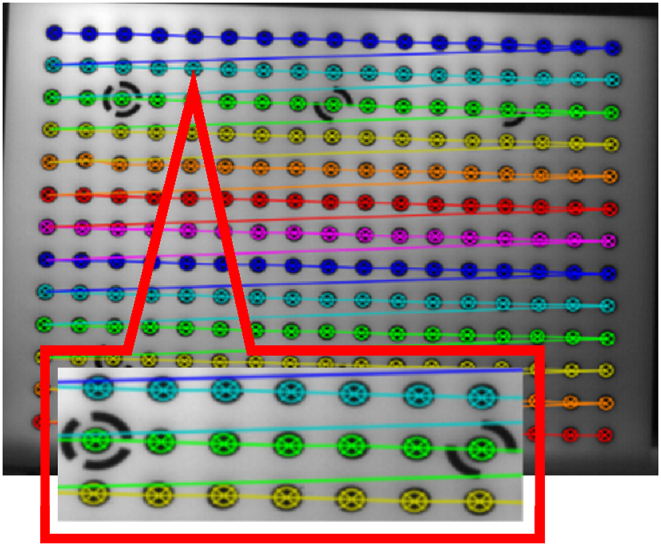
Fitting and extracting the targets using the HT algorithm. (Colored).

Geometric calibration of the thermal infrared sensor was done using the calibration pattern of a rectangular plate with hollow circles. The circle targets with a diameter of 12 mm each were arranged symmetrically in 13 rows and 17 columns. The distances between the centers of the circle targets were set at 24 mm. The six circles would serve as coded targets for determining the orientation and position of the targets in the various images of the calibration pattern. Hollow circles would provide for temperature differences, which could result in contrast on the thermal calibration pattern plate. As a result, this would improve the accuracy and quality of the HT algorithm in detecting the centers of circles in two-dimensional space. In this context, a passive source (heating radiator) applied heat to a calibration pattern to reveal circles and increase contrast in thermal infrared images. Then, using a thermal infrared camera, convergent images from various orientations and angles were acquired for geometric calibration and parameter estimation. Fig. 7 illustrates the result of applying the HT algorithm to fit and extract the focal center of ellipse targets from the pattern used to perform geometric calibration of the thermal infrared camera.

After extracting the precise two-dimensional coordinates of the center of the target focal to establish a relationship between the two-dimensional coordinates of the targets in image space and the object space, data was fed into collinearity equations to estimate the geometric calibration parameters of the thermal infrared sensor. Finally, using collinearity equations, the geometric calibration parameters of the thermal infrared camera were calculated. Table 4 provides the numerical values and standard deviations for the estimated interior orientation parameters and lens distortions in pixels for the thermal infrared camera.

**Table 4:** Thermal infrared camera geometric calibration parameters.

Parameters	Initial values (mm)	Standard deviation (pixel)	Values (pixel)
*F*_*x*_	25.00	89.86	1508.67
*F*_*y*_	00.00	90.44	1511.01
*C*_*x*_	00.00	27.39	34.023
*C*_*y*_	00.00	23.88	19.95
*K*_*1*_	00.00	0.14	−0.13
*K*_*2*_	00.00	5.10	1.15
*K*_*3*_	00.00	51.48	−29.88
*P*_*1*_	00.00	5.79 e−03	−0.001
*P*_*2*_	00.00	3.24 e−03	0.004

In Table 4, the numerical values and mean standard deviation of each calibration parameter are reported in pixels measurement units for the 13 images acquired from pattern calibration using the proposed method. The parameters *F*_*x*_ and *F*_*y*_ in the above table represent the focal lengths along the *x* and *y* axes, the *C*_*x*_ and *C*_*y*_ parameters stand for the principal point coordinates, the parameters *K*_*1*_, *K*_*2*_, and *K*_*3*_ indicate the lens’s radial distortions, and the parameters *P*_*1*_ and *P*_*2*_ show the thermal infrared camera’s tangential distortions. The results of computing calibration parameters are reflected in Table 4, where their means and standard deviations are compared with the thermal infrared camera’s original values as a reference.

**Fig. 8:**
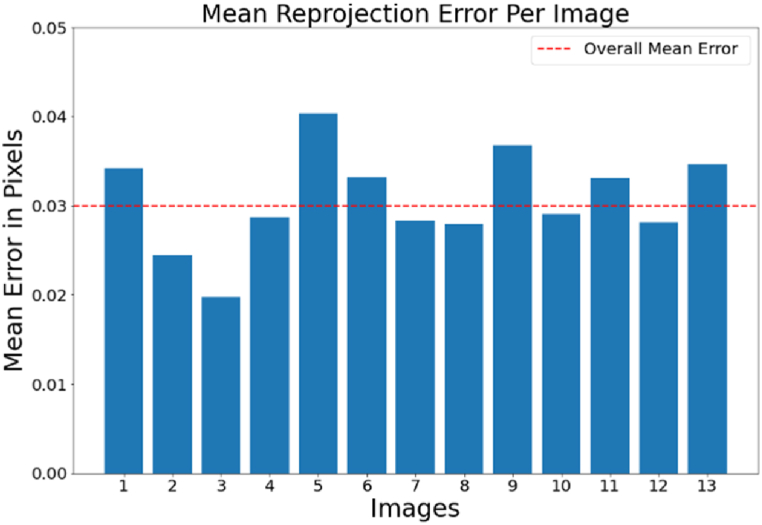
Illustrates the mean reprojection error for each image criterion. (Colored).

Then, the Mean Reprojection Error per image (MRE) was used to assess the accuracy of the geometric calibration of the thermal infrared camera. The MRE per Image of the geometric calibration of the thermal infrared camera is shown in Fig. 8.

The horizontal axis in Fig. 8 indicates the number of images; whereas, the vertical axis represents the reprojection error measured in terms of pixels for each image. As a result, the value of 0.03 pixel was estimated for 13 images collected from the calibration pattern and 221 targets using the mean reprojection error per image criteria.

### 3.2 Keyframes extraction

In this paper, contrast enhancement of thermal infrared images was performed just to overcome the low contrast and poor texture of thermal infrared frames, to avoid frame sequence disruption, and to increase the extractable features for verifying standard baseline and keyframes extraction. To generate a dense thermal infrared point cloud, after keyframes extraction, the raw keyframes were employed before the contrast enhancement stage.

**Fig. 9:**
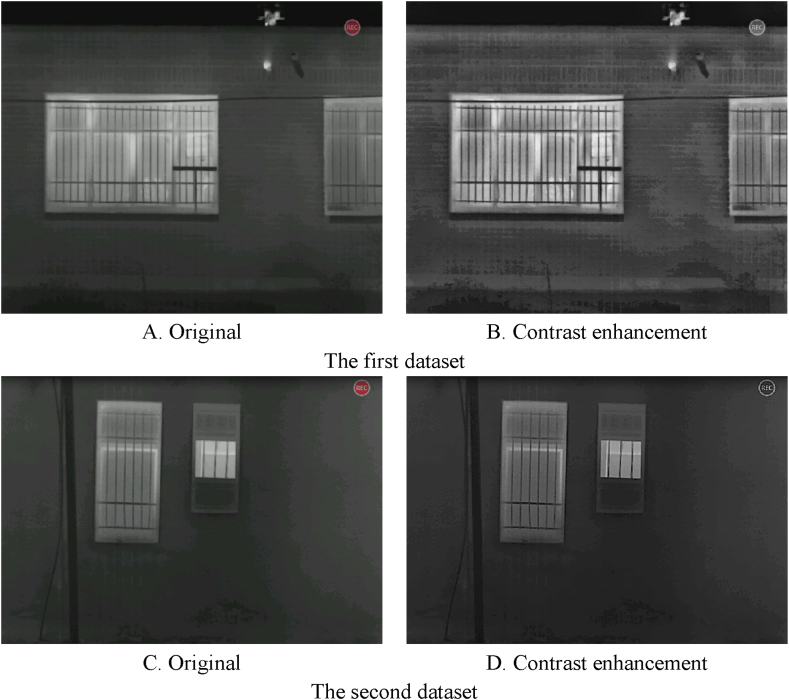
The results of contrast enhancement on thermal infrared images. (Colored).

The multiscale top-hat transform approach was utilized in this research to enhance the contrast of thermal infrared images. In this method, two structural elements, proportional geometric and flat, were employed, and as a result, this would improve the detail and entropy of the thermal infrared image. The outcome of the multiscale top-hat transform approach for thermal infrared frame contrast enhancement is depicted in Fig. 9.

Based on Fig. 9(A–D), after performing contrast enhancement processing using the multiscale top-hat transform approach on both thermal infrared image datasets, the thermal infrared image’s contrast was enhanced.

**Fig. 10:**
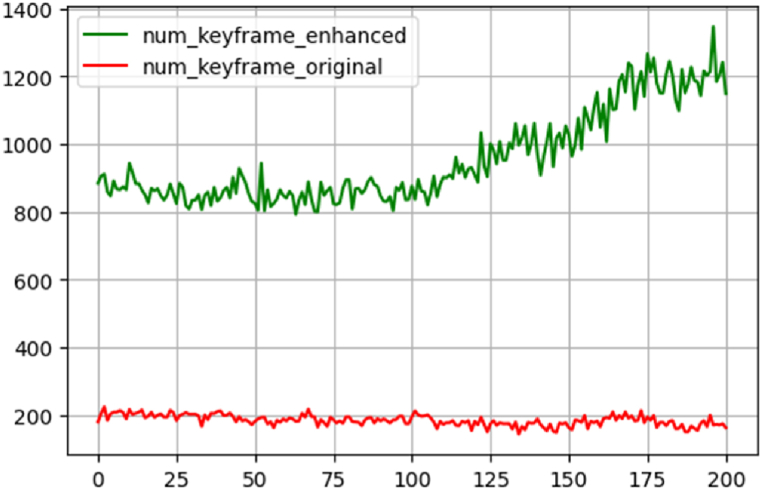
Shows The effect of contrast enhancement. (Colored).

Because of the low rate of features extracted from thermal infrared frames during the standard baseline check step, the overlap condition of 0.6–0.8 was not met, and the keyframes extraction sequence was cut. In this study, the contrast enhancement step was done solely to address the difficulty of low contrast and poor texture in thermal infrared pictures. The effect of contrast enhancement on the number of features extracted in the initial 200 frames is displayed in Fig. 10.

Based on the results shown in Fig. 10, enhancing contrast increases the number of features extracted from thermal infrared frames. The red graph depicts the extracted features from thermal infrared frames before contrast enhancement; whereas, the green chart displays the extracted features after contrast enhancement.

In the present study, the density and value of the point cloud reprojection error were used to evaluate the performance of keyframes extraction in generating the thermal infrared point cloud. Keyframes are effective in producing three-dimensional coordinates because of their strong geometry and high radiometric quality.

In the present work, the method for keyframe extracting was developed in Python by utilizing the OpenCV package. Therefore, for this purpose, the initial frame of the thermal infrared video file was extracted and entered the contrast enhancement stage in the first step. To continue, by determining the best threshold, blur frames were removed from the video frame dataset using the FFT metric to improve the geometric and radiometric accuracy of the dense thermal infrared point cloud generated. After inspecting the frames for radiometric quality, 853 blur frames were detected and eliminated from the first dataset with 1957 initial frames, and 214 blur frames were removed from the second dataset with 907 initial frames.

**Fig. 11:**
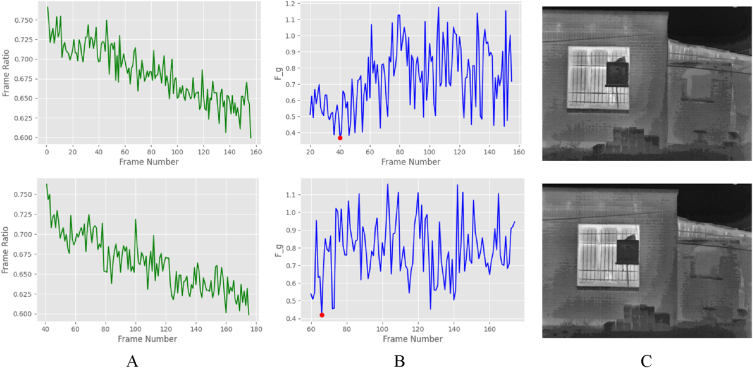
Displays the extracted keyframes. (Colored).

Then, using Eq. (5), the standard baseline between high-quality radiometric frames was determined. Frames with a typical overlap baseline falling between the higher and lower thresholds (0.6–0.8) were considered to have a normal overlap baseline. It then proceeded to the next stage, evaluating degeneracy conditions using the optimal GRIC criterion. The GRIC condition was used to analyze the degeneracy between pairs of frames. To this end, the GRIC was calculated by estimating the fundamental and homography matrix between pairs of frames using the RANSAC algorithm. Eq. (6) was then used to estimate the numerical value of the GRIC for the fundamental matrix as well as the homography matrix. Finally, using Eq. (7), the frame was extracted as the keyframe in which the fundamental matrix model between the two frames would have the smallest value compared with the homography matrix model. Fig. 11 illustrates the results of calculating the proportion of key points *R*_*c*_ and extracting keyframes using the GRIC criterion for two keyframes of the first dataset.

The outcomes of the keyframes extraction implementation are depicted in Fig. 11. Column A shows the coefficient of the proportion of *R*_*C*_ key points. Column B shows keyframes extraction using the GRIC criterion to compare the fundamental matrix with the homography matrix and select the fundamental matrix minimum value as the keyframe, and column C shows the extracted keyframe.

### 3.3 Thermal infrared and visible point cloud generation

Thermal infrared and visible point clouds were generated using image-based methods such as SfM and MVS. As a result, the stages for producing point clouds were performed independently for thermal infrared and visible data using SfM and MVS algorithms.

Thermal infrared and visible data with identical flight characteristics were collected from a distance of 11 m from the facade of the examined building. The numbers of visible images captured by a UAV for the first and second datasets were 46 and 24, respectively. Also, to generate a dense thermal infrared point cloud, raw keyframes were used before the contrast enhancement step. Then to explore the influence of keyframes extraction on generating a dense thermal infrared point cloud from video data, the criteria for evaluating the output point cloud and the value of reprojection error for both datasets were considered. In this regard, increasing the density and minimizing the reprojection error would indicate the optimal effect of keyframes extraction to produce a thermal infrared point cloud.

**Fig. 12:**
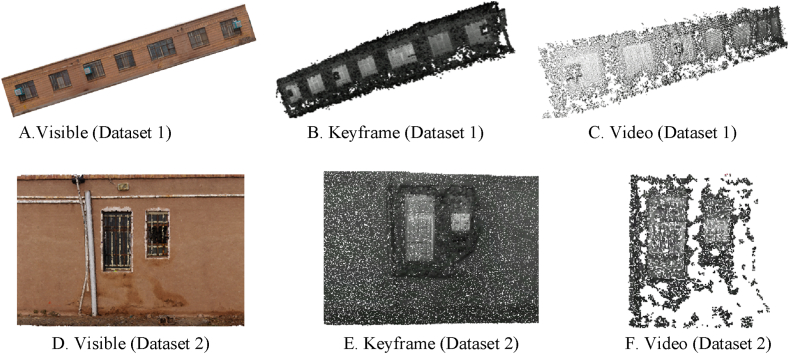
Displays the first and second dataset’s output point clouds (visible, thermal infrared video, and keyframes). (Colored).

Fig. 12 illustrates the output of the dense point cloud generation process using visible and thermal infrared images for video data utilization mode, and keyframes for the first and second datasets.

Sections (A–C) of Fig. 12 illustrate the output of the point cloud generated for the first dataset using visible data, thermal infrared video, and keyframes, while Sections (D–F) depicts the output of the point cloud generated for the second dataset using visible data, thermal infrared video, and keyframes. The output results of dense point cloud reconstruction for visible and thermal infrared data (in both video and keyframe modes) are provided in Table 5, together with the amount of reprojection error and Ground Sampling Distance (GSD) for both datasets.

**Table 5:** Dense visible and thermal infrared point cloud generation results based on density and reprojection error for both datasets.

Data Type	Density of Point Cloud (points per square meter)	Reprojection Error (pixels)	GSD (cm)
Visible (Dataset 1)	13,410,978	0.87	0.12
Visible (Dataset 2)	5,428,479	1.02	0.12
Thermal (Dataset 1-Video)	800,106	0.83	0.75
Thermal (Dataset 1-Keyframe)	1,779,067	0.41	0.75
Thermal (Dataset 2-Video)	16,641	1.31	0.75
Thermal (Dataset 2-Keyframe)	178,524	0.66	0.75

In Table 5, the results of the numerical evaluation of the density of the generated dense point cloud and the reprojection error values for visible images and thermal infrared video keyframes for both datasets are presented. As presented in the table, the results indicated that the employment of thermal infrared keyframes would improve the density of the generated point cloud and would decrease its reprojection error. The best values for increasing the density and decreasing the reprojection error of a thermal infrared point cloud produced using a bold pen are presented in Table 5.

### 3.4 Thermal infrared and visible point cloud fusion

**Fig. 13:**
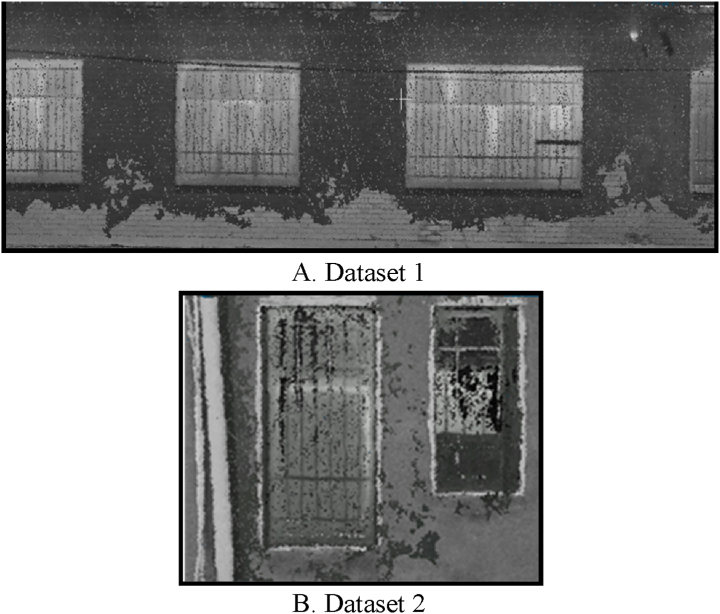
Displays the output of the visible and thermal infrared point clouds for both datasets. (Colored).

After generating thermal infrared and visible point clouds, the fusion of visible and thermal infrared point clouds was performed to overcome the inferior density thermal infrared point cloud caused by the low spatial resolution of thermal infrared frames. The ICP algorithm was adopted in this research to map the radiometric information of the nearest three-dimensional point in the thermal infrared point cloud onto its equivalent point in the visible point cloud. Thus the resulting point cloud had a high density and level of detail with thermal information. Fig. 13 illustrates the fusion of the visible and thermal infrared point cloud datasets using the ICP algorithm. Meanwhile, Table 6 presents the density of the fused point cloud as a Root Mean Square (RMS) value.

**Table 6:** The density of the fused point cloud based on the RMS value of the two datasets.

Dataset	Density of Point Cloud (points per square meter)	Root Mean Square	Density Ratio of Point Cloud
Dataset 1	13,700,232	0.0003	6 times
Dataset 2	817,767	0.0004	4 times

**Fig. 14:**
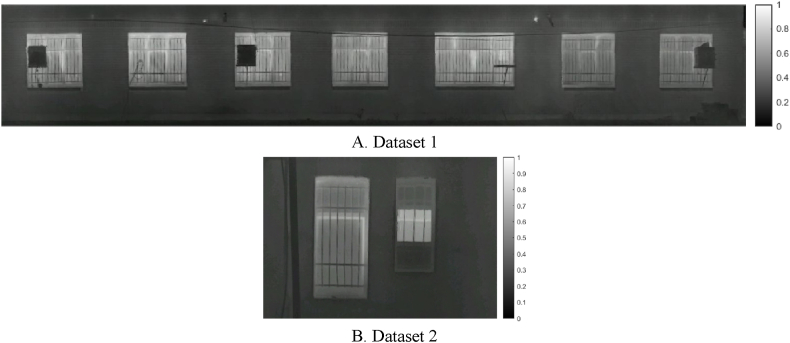
Orthophoto mosaic output for both datasets. (Colored).

The data in Table 6 indicate a sixfold and fourfold increase in the density of the fused point cloud for both datasets, using the RMS criterion. Finally, an orthophoto mosaic was generated using a point cloud that had been fused. The orthophoto mosaic output for both datasets is shown in Fig. 14. In Fig. 14, the brighter sections of the orthophoto mosaic indicate regions on the building facade with higher temperatures and thermal leakage points of.

### 3.5 Extraction of components from a building’s facade

**Fig. 15:**
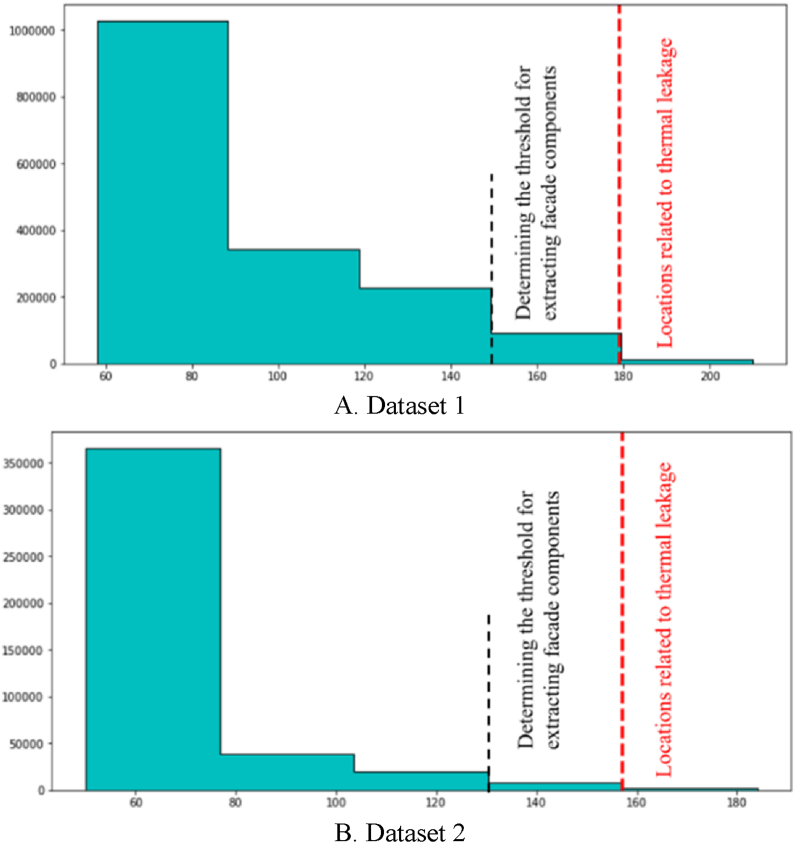
Set thresholds for both datasets. (Colored).

In this stage, to extract the components of the building facade, the orthophoto mosaic generated in the previous stage was employed. The orthophoto mosaic histogram illustrates the distribution of grayscale values corresponding to each pixel. First, the Python programming language was used to extract the building facade components. Next, the frequency distribution of grayscale values was computed to generate the orthophoto mosaic temperature histogram. Afterward, two thresholds depending on the temperature distribution were employed to extract the edge pixels and the components and locations in the building facade that may have had thermal leakage. As a result, the orthophoto mosaic histogram was experimentally separated into five distinct categories with identical temperature distributions to calculate the two thresholds. Fig. 15 illustrates the thresholds computed using the histogram temperature distribution for extracting components and locations with the potential for thermal leakage in the building facade for both datasets.

**Fig. 16:**
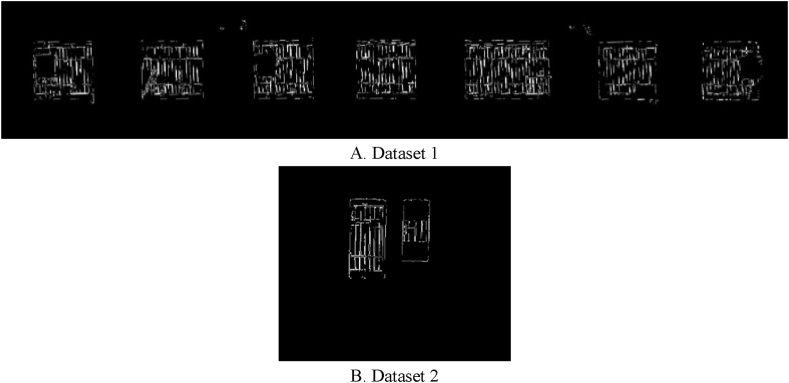
The initial edge extraction for both datasets. (Colored).

**Fig. 17:**
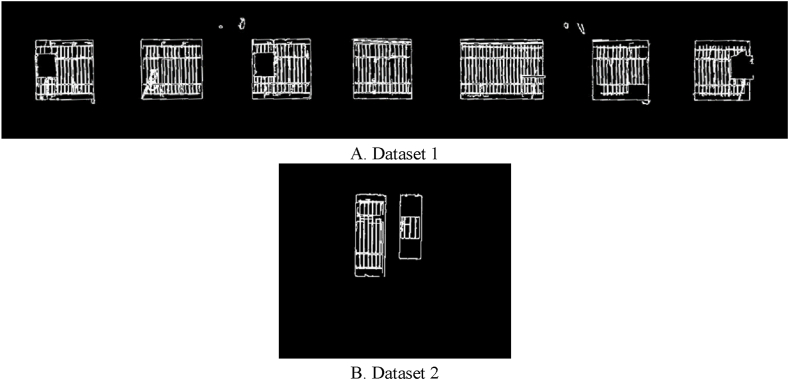
Displays the dilation operator for the two datasets. (Colored).

As shown in Fig. 15, the threshold associated with the determination of thermal leakage locations is the evaluation of candidate areas related to thermal leakage; whereas, the threshold associated with extracting facade components is the assessment of sufficient temperature changes in candidate areas. The next step was to extract orthophoto mosaic edges using the two thresholds defined as the optimal threshold values. The initial edges were extracted in this paper using the canny edge extraction operator and the Python programming language. The initial edge extraction results from the orthophoto mosaic are shown in Fig. 16. Fig. 16 illustrates the initial edge extraction results for both datasets to extract components and locations with the potential for thermal leakage in the building facade. After extracting the primary edges, they were improved using the dilation morphology operator. In Fig. 17, the dilation operator’s outcomes for improving the initial edges for both datasets using the Python programming language are schematically depicted. Based on Fig. 17, the dilation operator enhanced the image’s binary regions. It also removed holes from the binary image, resulting in the repair of incorrect (damaged) edges.

### 3.6 Thermal leakage map generation

**Fig. 18:**
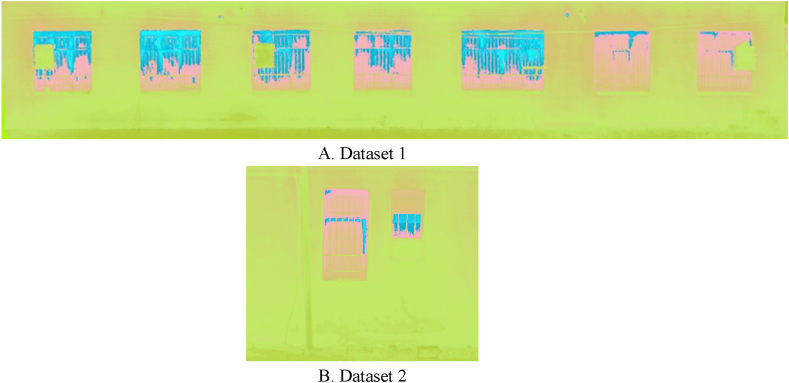
Conversion of orthophoto mosaic thermal infrared color space to HSV color space. (Colored). (For interpretation of the references to color in this figure legend, the reader is referred to the Web version of this article.)

In this paper, thermal infrared orthophoto mosaics and the Region-Growing segmentation algorithm were utilized to extract and determine the locations of thermal leakage in the building facade. In this regard, it seemed necessary to execute a significant pre-processing phase, including color space transformation, to more precisely pinpoint the regions of thermal leakage. Since the saturation parameter distribution in the HSV color space would allow for the extraction of areas with higher temperatures, orthophoto mosaic pixels in the range of thermal infrared grayscale values were converted to the HSV color space using the Python programming language. In Fig. 18, the conversion of orthophoto mosaic thermal infrared color space to HSV color space is displayed for both datasets.

In Fig. 18, the blue color sections represent areas of higher temperature and thermal leakage locations in the orthophoto mosaic output in the HSV color space. To segment the pixels and areas associated with thermal leakage in the examined building’s facade, the Region-Growing segmentation method was employed. In the present study, the proposed technique was developed utilizing the Python programming language. In this approach, seed pixels are required to segment thermal leakage areas. Therefore, given that the purpose was to segment thermal leakage locations in the building’s facade and produce a thermal leakage map, the seed pixels were chosen from the saturation parameter in the HSV space. The following steps describe how to implement the Region-Growing segmentation algorithm:

• The seed pixel is chosen based on the maximum intensity of grayscale values and is compared to nearby pixels using the criterion of grayscale value intensity similarity.
• Each segment increases the size of the original piece by g similar surrounding pixels starting with the seed pixel.
• When a segment’s growth is complete, a new seed pixel is chosen that is not yet associated with any component, and the process is repeated from the beginning.
• This is repeated until all pixels have been assigned to a segment.

**Fig. 19:**
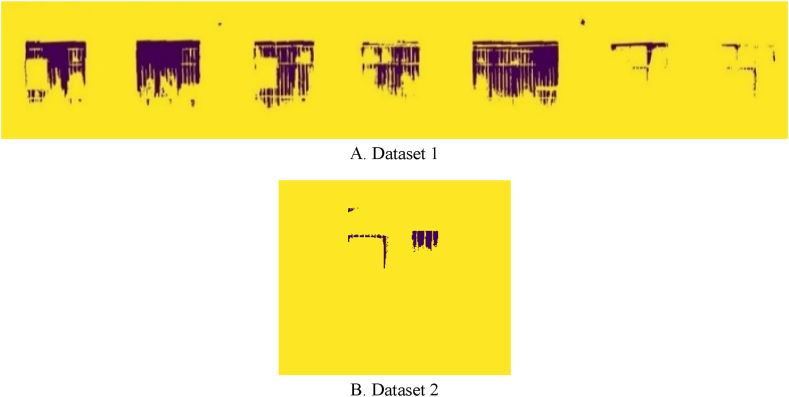
Demonstration of the Thermal leakage location using the Region-Growing segmentation algorithm for both datasets. (Colored).

**Fig. 20:**
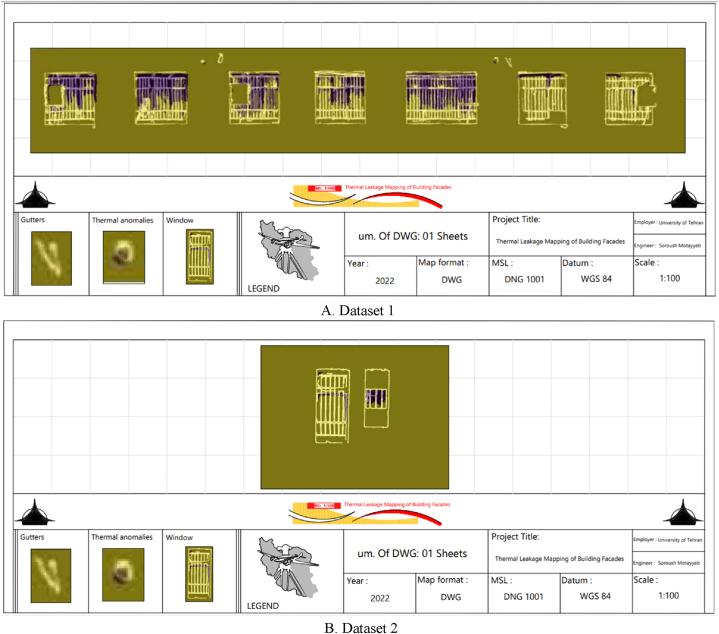
Thermal leakage map of building’s facade. (Colored).

The Region-Growing algorithm’s segmentation results for thermal leakage areas map on orthophoto mosaics are shown in Fig. 19. Here, the purple color segments in the output image represent the locations of the building facade’s thermal leakage. Fig. 20 depicts the thermal leakage map of the building facades for both datasets. Finally, as shown in Fig. 20, the thermal leakage map of the building facade is generated by mapping the output of the component extraction procedures and identifying the thermal leakage locations relative to one another.

#### 3.6.1 Evaluating the results of determining the locations of thermal leakage

Two criteria for measuring precision and recall were used in this study to evaluate the proposed algorithm for determining the locations of thermal leakage. Precision and recall criteria are expressed as values between 0 and 1. The closer the output results are to 1 or 100%, the more acceptable the recommended method’s performance will be.

Moreover, two orthophoto mosaics in HSV color space and segmented were to execute and assess the outcomes. It is worth noting that the orthophoto mosaic in HSV color space was chosen as a reference for evaluating and comparing thermal leakage locations. Therefore, to analysis the results, it was necessary to normalize the grayscale values of pixels in the HSV and the segmented orthophoto mosaic. Then using the Python programming language, the two orthophoto mosaics were compared pixel by pixel. Finally, the output matrix was obtained as a logical matrix following the comparison. Then the precision and recall evaluation criteria were calculated as follows:

• The values with a numerical value equal to one in the logical matrix represent the variable *T*_*p*_*.*
• The values with a numerical value equal to zero in the logical matrix represent the variable *F*_*p*_*.*

To determine the *F*_*n*_ variable, the obtained numerical value of the *T*_*p*_ variable must be deducted from the total number of pixels associated with thermal leakage locations in the HSV orthophoto mosaic.

In Table 7, the proposed approach for the thermal leakage areas map on the facade of the investigated building is evaluated using the precision and recall criteria for both datasets. The results of determining the areas of thermal leakage using these criteria were examined by a thermography expert.

**Table 7:** Evaluation of the proposed method for thermal leakage areas map.

Dataset	*Tp* (pixel)	*Fp* (pixel)	*Fn* (pixel)	Precision (%)	Recall (%)
Dataset 1	107,032	10,932	14,668	90	87
Dataset 2	3490	452	4842	88	87

## 4 Discussion

In this paper, after geometric calibration of the thermal infrared camera with a mean reprojection error of 0.03 pixels, contrast enhancement and keyframes extraction from the thermal infrared video were performed to improve the geometric precision of the point cloud and reduce the volume of aerial triangulation calculations. Based on the findings reported in Table 5, keyframe extraction enhanced the density of the thermal infrared point cloud by 2–11 times while it decreased the reprojection error by two times.

In this study, to overcome the thermal infrared camera limitations (low spatial resolution, long focal length, and poor texture), the dense thermal infrared and visible spars point clouds were fused at the point cloud level using the ICP algorithm. As a result, the fused point cloud had a high spatial resolution and density, as well as thermal information. Fusion of the point clouds was estimated for both datasets using MSR values of 0.0003 and 0.0004, respectively, and the fused point clouds were found to be sixfold and fourfold denser than the initial thermal infrared point cloud, respectively.

Table 8, presents and analyses the outcomes of the proposed approach for orthophoto mosaics generated from the fusion of visible and thermal infrared point clouds, orthophoto mosaics resulting from the thermal infrared point cloud (OM-TIPC), and related research. In this regard, the results demonstrate an improvement in the proposed method’s accuracy in determining the locations of thermal leakage based on precision and recall evaluation criteria.

**Table 8:** Investigation methods of locating thermal leakage areas.

Methods	Precision (%)	Recall (%)
Kakillioglu et al. [32]	34	71
Rakha et al. [31]	76	74
Proposed method (OM-TIPC (Dataset 1))	62	65
Proposed method (OM-TIPC (Dataset 2))	66	65
Proposed method (Dataset 1)	**90**	**87**
Proposed method (Dataset 2)	**88**	**87**

**Fig. 21:**
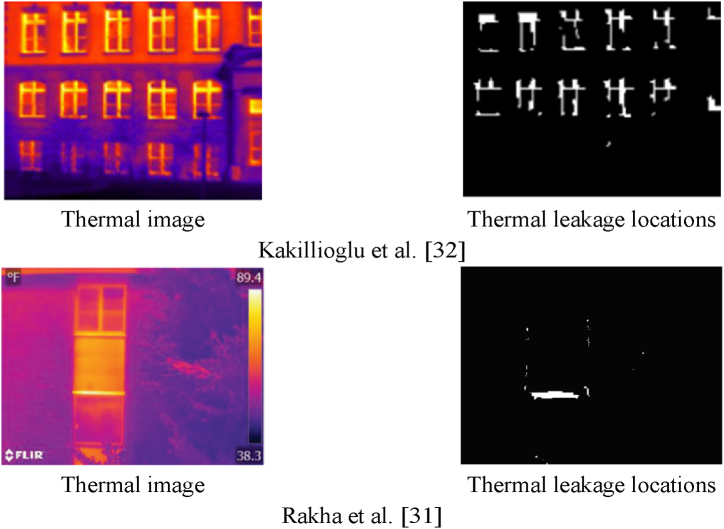
The display of the segmentation results to identify areas of thermal leakage. (Colored).

Based on the precision and recall evaluation criteria for both datasets, the proposed approach improved the accuracy of determining thermal leakage locations, as shown in Table 8. Fig. 21 depicts the visual results for locating thermal leakage relevant to the purpose of the study.

The following sections address the proposed method’s points and advantages, as well as its accomplishments and purpose:

• Due to the employment of an aerial platform equipped with inexpensive thermal infrared cameras, it is possible to appropriate flight operations according to the geometry of the building, which can also be highly effective for inspecting buildings with greater heights. In this regard, photogrammetry can be used to conduct periodic energy audits of facilities and to create large-scale maps of building thermal leakage.
• Generally, photogrammetry eliminate the need to visualize energy loss, determine thermal leakage locations, and estimate the amount of energy loss through the use of images taken by UAVs from different views of the building and products of photogrammetry.
• As the research assumes to consider areas with scattered urban or industrial buildings, we can attest that in the proposed method, the human aspect engaged in thermography is omitted in terms of participation and responsibility. In this respect, using UAV photogrammetry products and automated computer vision algorithms to study the energy condition of the structure and to pinpoint areas of thermal leakage can boost speed, and accuracy and decrease costs and time. As a result, speed and accuracy will increase, while the price and time associated with finding the areas of thermal leakage will decrease.

## 5 Conclusion

In recent years, enhancing building energy efficiency to maximize the use of energy production resources while minimizing costs has become a critical concern in the construction industry. The present research proposed a method to increase the energy efficiency of buildings by extracting building facade components and determining a thermal leakage map. In this work, the fusion of two data sets including thermal infrared and visible images captured by the UAV was used. According to the proposed method, thermal leakage locations on orthophoto mosaic were investigated utilizing precision and recall assessment criteria. The precision and recall evaluation criteria for the first data set were equal to 90% and 87%, and these criteria were calculated as 87% and 88% for the second data set, respectively. On the whole, the results demonstrate that the suggested method can improve the accuracy of locating thermal leakage areas in a building facade.

The following are some recommendations for future research on locating leakage areas and thermal leakage map generation of the buildings:

• Utilizing thermal data that has been geotagged with GPS/IMU (Inertial Measurement Unit) helps improve the accuracy of point cloud generation and fusing of visible and thermal infrared point clouds.
• To determine and segment the areas of thermal leakage, recent deep learning-based segmentation approaches can be compared to the method suggested in this research.
• As a follow-up to this research, due to the requirement to improve energy efficiency, the production of a thermal leakage map of the whole building’s facade, including the walls and ceilings, can be done.
• Depending on a research premise on data collection from places with scattered or industrial structures, complicated urban areas could be investigated for future research.

## Author contribution statement

Farzaneh Dadrass Javan: Conceived and designed the experiments; Analyzed and interpreted the data; Wrote the paper.

Soroush Motayyeb: Performed the experiments; Analyzed and interpreted the data; Wrote the paper.

Farhad Samadzadegan: Conceived and designed the experiments; Contributed reagents, materials, analysis tools or data.

Hamidreza Hosseinpour: Performed the experiments; Wrote the paper.

## Funding statement

This research did not receive any specific grant from funding agencies in the public, commercial, or not-for-profit sectors.

## Data availability statement

The data that has been used is confidential.

## Declaration of interest’s statement

The authors declare no conflict of interest.

## Footnotes

1 Retrieved from https://www.explainthatstuff.com/.
